# Bottle beam generation from a frequency-doubled Nd:YVO_4_ laser

**DOI:** 10.1038/s41598-018-34783-z

**Published:** 2018-11-08

**Authors:** J. C. Tung, Y. Y. Ma, K. Miyamoto, Y. F. Chen, T. Omatsu

**Affiliations:** 1 Graduate School of Engineering, Chiba University, 1-33 Yayoi-cho, Inage-ku, Chiba 263-8522 Japan; 2 Molecular Chirality Research Center, Chiba University, 1-33, Yayoi-cho, Inage-ku, Chiba 263-8522 Japan; 3 Department of Electrophysics, National Chiao Tung University, 1001, Ta-Hsueh Rd., Hsinchu, 30010 Taiwan

## Abstract

We demonstrate, for the first time, the direct generation of a bottle beam with a well-isolated three-dimensional zero-intensity dark core (high potential barrier) from a compact intracavity frequency-doubled Nd:YVO_4_ laser with a nearly hemispherical cavity. We also numerically calculate the physical properties of the generated bottle beam using a coherent superposition of a series of frequency-locked Laguerre–Gaussian modes.

## Introduction

Optical bottle beams^1–3^, which possess a central zero-intensity core surrounded by three-dimensional (3D) bright regions, provide numerous applications, such as optical tweezers for atom trapping and light-absorptive particle guiding^4–11^, fluorescence microscopes with high 3D spatial resolution^12,13^ and the cloaking of reflection, scattering or transmission from an object^14,15^. Such applications rely heavily on the generation of bottle beams with a well-isolated 3D zero-intensity dark core, i.e. a high potential barrier.

To date, several techniques for bottle beam generation have been proposed, for example, the use of computer-generated digital holograms^1,8–11^, in which several Laguerre–Gaussian (LG) modes with zero azimuthal and non-zero radial indices, i.e. radial LG modes, are coupled destructively or constructively in the far field. Such digital holograms can potentially be used to create desired bottle beams with arbitrary amplitude and phase distributions; however, they require rather complex calculations for designing the bottle beams.

Bottle beams can also be produced using a solid-state laser with near-degenerate cavities^16^, axicon-lens focusing of Bessel beams^17,18^, conical refraction of light in a biaxial crystal^19^, stress-engineered optical elements^20^, or frequency-doubled (or sum-frequency) self-Raman lasers^21,22^; however, they generate only bottle beams with a relatively low potential barrier. It remains difficult to generate bottle beams with a high potential barrier using only conventional optical devices.

In recent years, the direct generation of various radial LG modes from a tightly pumped solid-state laser with a nearly hemispherical resonator configuration, in which the higher-order radial LG modes are allowed to lase as the pump power is increased, has been demonstrated^23^. Furthermore, it has been reported that the second-harmonic generation (SHG) of a radial LG mode can coherently superimpose several radial LG modes to produce bottle beams^24^.

In the present study, we propose a new approach to generate bottle beams, which we refer to as a bottle beam laser resonator, in which a tightly pumped nearly hemispherical Nd:YVO_4_ laser resonator is combined with an intra-cavity SHG configuration. Using this design, we demonstrate the direct generation of a green bottle beam with a rather higher potential barrier compared with those of the previous reports^16,24^. The generated bottle beam exhibits unique 3D beam propagation, namely, central bright spots in the near and far fields, and a dark core in the intermediate region between the near and far fields. We also conduct a theoretical analysis of the 3D beam propagation of the generated bottle beam.

## Results

### LG mode generation

**Figure 1:**
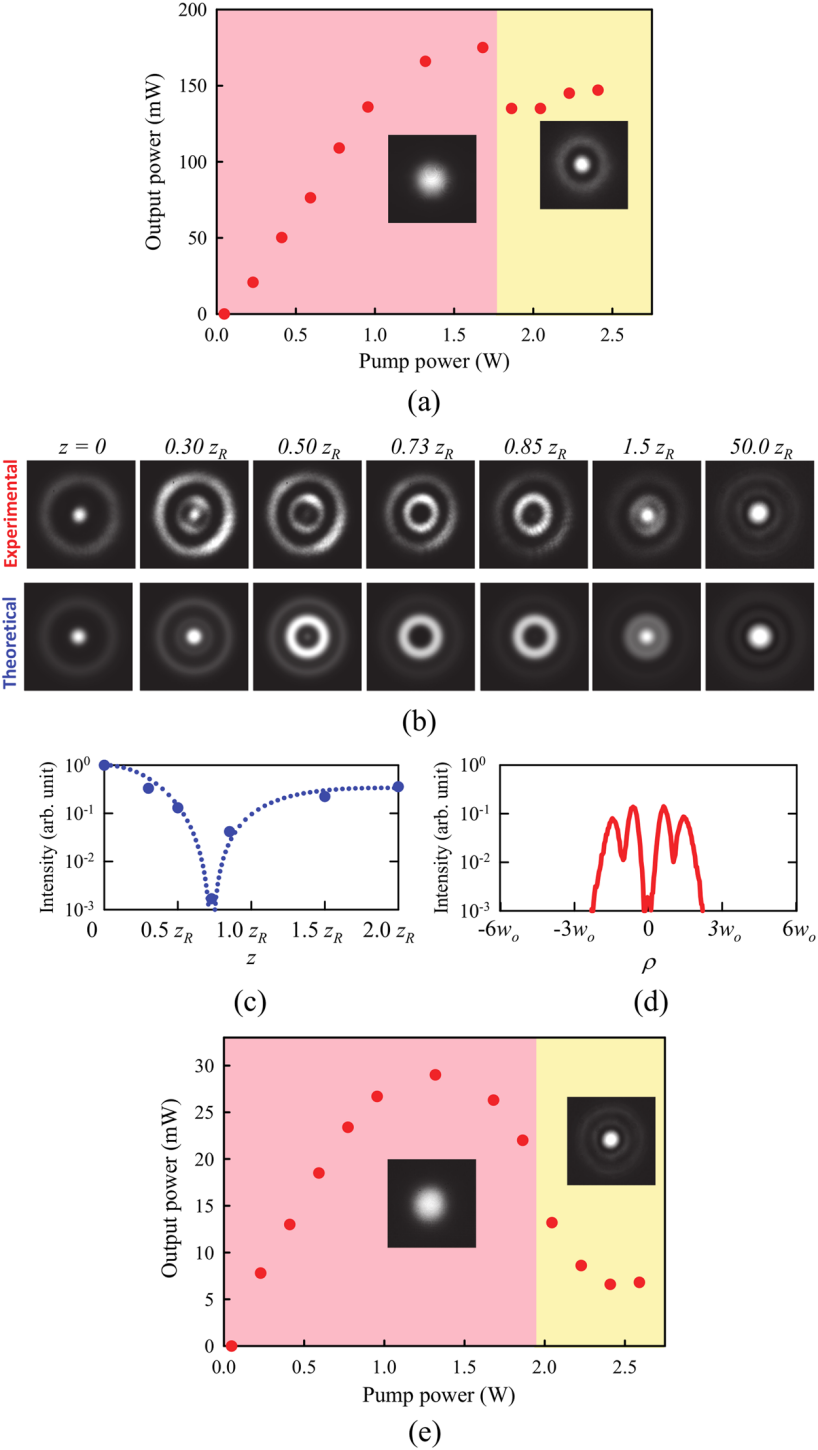
(**a**) Experimental output powers and fundamental lasing modes at various pump powers. Insets show spatial forms of fundamental lasing modes. Fundamental output lased at a Gaussian mode (1^st^-order LG mode) within a pump power of <1.75 W (>1.75 W). (**b**) Experimental (upper row) and calculated transverse spatial forms (lower row) of the bottle beam at different longitudinal positions. (**c**) Experimental intensity profiles of the bottle beam along the propagation and radial directions. (**d**) Experimental output power and second-harmonic far-field patterns at various pump powers. Insets show spatial forms of the second harmonics. The bottle beam was generated at the pump power of >1.75 W.

The output generated from our system exhibited a spatial form associated with a radial LG mode with a radial index of *p* = 0 or 1 within the pump power region of 0.05–1.75 W (red zone) or 1.75–2.40 W (yellow zone), as shown in Fig. 1(a), respectively. Notice that the output is also expected to include undesired low-order modes because of the frequency-locking effects between the transverse and longitudinal modes in a nearly hemispherical cavity^23^, e.g., the first-order radial LG mode includes a Gaussian mode as an impurity. Such transversely multi-mode operation plays an important role in manifesting a bottle beam with a high potential barrier by employing SHG. The output power was measured to be 130–150 mW within a pump power region of 1.75–2.40 W.

### Bottle beam generation

To efficiently perform the intra-cavity SHG, we replaced the 98% reflective output coupler with a high-reflection mirror for 1064 nm (R > 99.8%). A 1 mm KTiPO_4_ (KTP) crystal cut for type-II phase matching between 1064 nm and 532 nm was also placed near the output coupler. The distance, *d*, between the KTP and the output coupler was varied. A dichroic mirror with high transmissivity (T > 95%) for 1064 nm and high reflectivity (R > 99.8%) for 532 nm was used to remove the undesired fundamental beam. The second harmonics, i.e. the green beam, was delivered and imaged by two lenses with focal lengths of 125 mm and 400 mm onto a CCD camera mounted on a one-dimensional translation stage. When the KTP crystal was placed close enough (*d* ≈ 1.0 mm) to the flat output coupler, the second harmonics exhibited the typical physical characteristics of a bottle beam: central bright spots in the near and far fields, and a central dark core with a potential barrier in the intermediate between the near and far fields, i.e. at the longitudinal position, where *z*_*R*_ is the Rayleigh length (*z*_*R*_ ≈ 4.8 mm), as shown in the upper row of Fig. 1(b). The generated bottle beam also exhibited a well-isolated and narrow dark core surrounded by a relatively symmetric bright zone (i.e. potential wall) along the propagation and radial directions, thereby yielding a deep potential well, as shown in Fig. 1(c).

Such a bottle beam was attained in the pump power region of 2.0–2.4 W (yellow zone) in Fig. 1(d), and its output power was measured to be 13.2 mW at a pump power of 2.0 W. Even a milliwatt-level bottle beam should be useful for the aforementioned applications, in particular, applications toward various biological systems, micromachining, and medical applications when used in combination with a tightly focusing objective lens. Furthermore, it is possible to scale the output power provided by the system by optimizing the cavity design and the thickness of KTP crystal.

## Discussion

The radial-order LG mode Φ_*p*,*s*_ (*ρ*, *ϕ*, *z*, *φ*) with a transverse radial index *p* and a longitudinal mode index *s* is expressed in cylindrical coordinates (*ρ*, *ϕ*, *z*) as

,1 $${{\rm{\Phi }}}_{p,s}(\rho ,\varphi ,z,\phi )=\sqrt{\frac{1}{\pi }}\frac{1}{w(z)}{L}_{p}(\frac{2{\rho }^{2}}{{w}^{2}(z)}){e}^{-{\rho }^{2}/{w}^{2}(z)}{e}^{-i{k}_{p,s}\tilde{z}}{e}^{i(2p+1)[{\theta }_{G}(z)+\phi ],}$$

where

2 $$\tilde{z}=z+[z{\rho }^{2}/2({z}^{2}+{z}_{R}^{2})],$$

3 $$w(z)={w}_{o}\sqrt{1+{(z/{z}_{R})}^{2}},$$

4 $${\theta }_{G}(z)=\,{\tan }^{-1}(z/{z}_{R}),$$

and

5 $${z}_{R}=\pi {w}_{o}^{2}/\lambda .$$

Here, *L*_*p*_(·) is the *p*^th^-order Laguerre polynomial, *w*_*o*_ is the beam radius at the waist, *λ* is the wavelength, and *φ* is the relative phase among the various LG modes at *z* = 0. In addition, *k*_*p*,*s*_ is the wavenumber given by ${k}_{p,s}=[s+2p({\rm{\Delta }}{f}_{T}/{\rm{\Delta }}{f}_{L})]\pi /L$, where *L* is the effective cavity length, and Δ*f*_*T*_ = *c*/2*L* and Δ*f*_*T*_ are the longitudinal and transverse mode spacings, respectively. A hemispherical cavity configuration with a ratio of Δ*f*_*L*_/Δ*f*_*T*_ ≈ 2 induces longitudinal-transverse mode coupling, which encourages frequency locking among different longitudinal and transverse modes^25^. Thus, the fundamental lasing mode *up*(*ρ*, *ϕ*, *z*, *φ*) is expressed as a coherent superposition of frequency-degenerate LG modes ${{\rm{\Phi }}}_{q,N-q}(\rho ,\varphi ,z,\phi )$ with *q* = 0, 1, 2, …™, *p* and *N* ≈ 2*L*/*λ* (*N* ≫ 1) as follows:

6 $${u}_{p}(\rho ,\varphi ,z,\phi )=\sum _{q=0}^{p}{a}_{q}\,\,{{\rm{\Phi }}}_{q,N-q}(\rho ,\varphi ,z,\phi ),$$

where *a*_*q*_ is the amplitude of the radial-order LG mode with radial index *q* and satisfies the summation $\sum _{q}{a}_{q}=1$. The resulting second-harmonic electric field Ψ_*p*_(*ρ*, *ϕ*, *z*, *φ*) can be expressed as

7 $${{\rm{\Psi }}}_{p}(\rho ,\varphi ,z,\phi )=\eta {[\sum _{q=0}^{p}{a}_{q}{{\rm{\Phi }}}_{q,N-q}(\rho ,\varphi ,z,\phi )]}^{2},$$

where *η* is a constant related to the effective second-harmonic conversion efficiency.

The second-harmonic electric field with *p* = 1 is given by

8 $${{\rm{\Psi }}}_{1}(\rho ,\varphi ,z,\phi )=\eta {[{a}_{0}{{\rm{\Phi }}}_{0,N}(\rho ,\varphi ,z,\phi )+{a}_{1}{{\rm{\Phi }}}_{1,N-1}(\rho ,\varphi ,z,\phi )]}^{2}.$$

The second-harmonic lasing mode can, thus, be expressed by

9 $${{\rm{\Psi }}}_{1}(\rho ,\varphi ,z,\phi )=\eta ^{\prime} [{b}_{0}\,{{\rm{\Phi }}^{\prime} }_{0,N}(\rho ,\varphi ,z,\phi )+{b}_{1}\,{{\rm{\Phi }}^{\prime} }_{1,N-1}(\rho ,\varphi ,z,\phi )+{b}_{2}\,{{\rm{\Phi }}^{\prime} }_{2,N-2}(\rho ,\varphi ,z,\phi )],$$

where ${b}_{0}={a}_{0}^{2}+{a}_{0}{a}_{1}{e}^{2i[{\theta }_{G}(z)+\phi ]}+({a}_{1}^{2}/2)\,{e}^{4i[{\theta }_{G}(z)+\phi ]}$, *b*_1_ = *a*_0_*a*_1_, and ${b}_{2}={a}_{1}^{2}/2$ (see Supplementary Information). The mode components *a*_0_ and *a*_1_ for the crystal placed close enough (*d *≈ 1.0 mm) to the flat output coupler were determined to be 0.29 and 0.71, respectively, so as to retrieve the experimental spatial forms at different longitudinal positions, as shown in the lower row of Fig. 1(b). The relative phase *φ* between the transverse and longitudinal modes was then assigned to be 0, owing to the extremely large acceptance bandwidth (~220 nm) of the 1 mm thick KTP crystal^26^. Also, notice that the non-zero relative phase *φ* should be taken into account as increasing *d*, i.e. as the nonlinear crystal is moved away from the output coupler.

**Figure 2:**
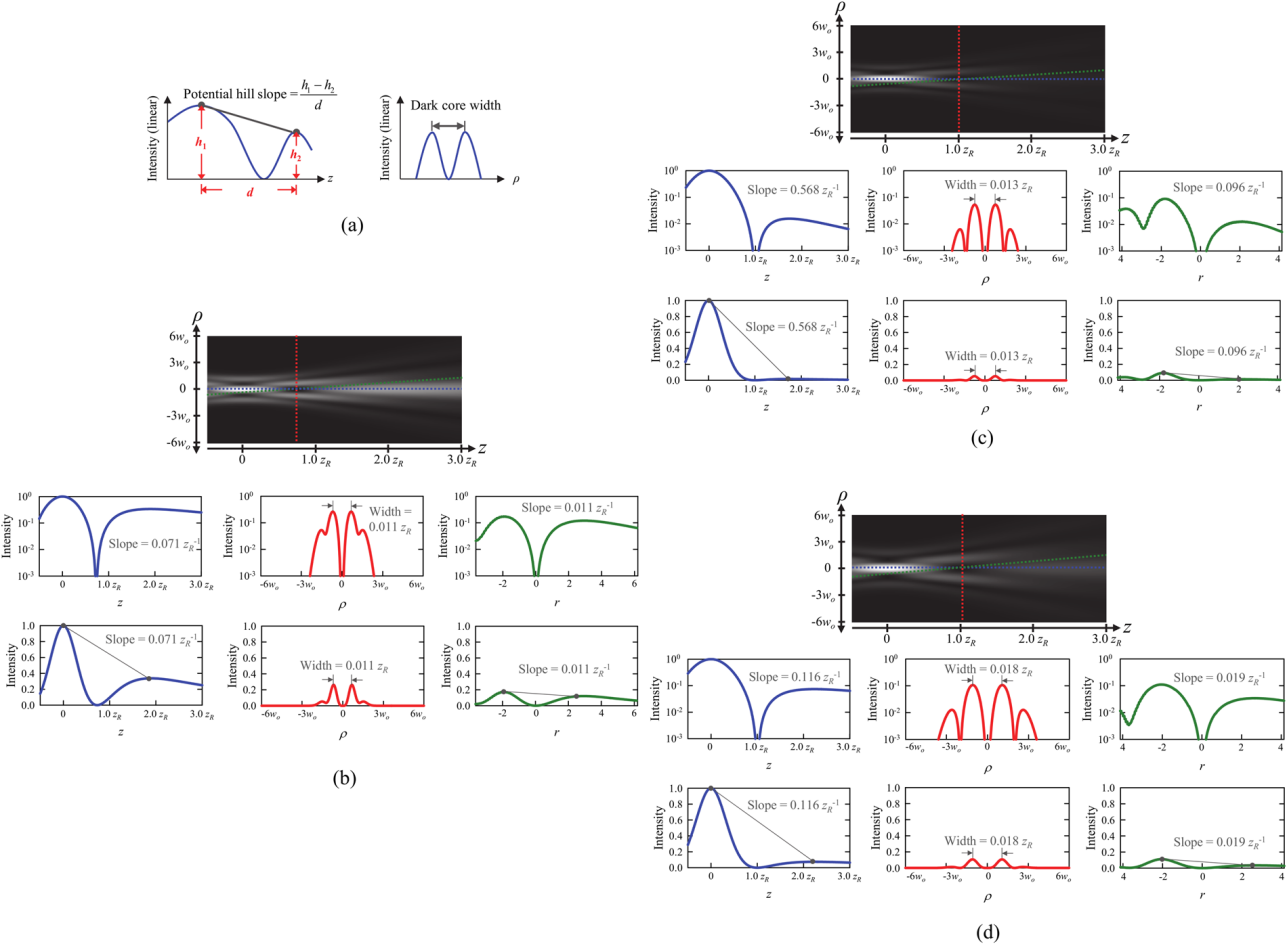
(**a**) Illustration of definitions of potential hill slope and dark core width. Calculated 2D intensity evolutions and corresponding intensity profiles along different directions (propagation, radial, and minimum intensity) for (**b**) the lower row of Fig. 1(b) (our proposed ‘bottle beam laser resonator), (**c**) external SHG of the first-order radial LG mode, and (**d**) coherent superposition of two LG modes with radial index *p* = 0 and 2.

Theoretical analyses agree well with the experimentally observed bottle beam properties, in which central bright spots appear in the near and far fields, and a central dark core with a high potential barrier is seen in the intermediate region between the near and far fields. The generated bottle beam also exhibits a well-isolated and narrow 3D dark core surrounded by a rather symmetric potential well along the propagation, radial, and potential valley directions (Fig. 2(a)). Such a bottle beam is generated by constructive or deconstructive interference of three LG modes with radial indices of 0–2 arising from Gouy-phase effects.

Bottle beams generated by external SHG of only the first-order radial LG mode^24^ (Fig. 2(b)) and coherent superposition of two LG modes with a radial index of *p* = 0 and 2^16^ (Fig. 2(c)), were simulated in the same way. In contrast to the above case, these show right descending asymmetry along the propagation and potential valley directions and a rather wide dark core along the radial direction. In fact, the bottle beam generated by our proposed method exhibits relatively low potential hill slopes, defined as the intensity difference of two maximal points divided by the distance between the maximal points along the propagation and potential valley directions. These were 0.071 *z*_*R*_^−1^ and 0.011 *z*_*R*_^−1^ along the propagation and potential valley directions and a narrow dark core width, defined as the interval between the maximum potential points along the radial direction, of 0.011 *z*_*R*_, compared with those (potential hill slope along the propagation direction of 0.116–0.568 *z*_*R*_^−1^, potential hill slope along the potential valley direction of 0.096–0.019 *z*_*R*_^−1^, and dark core width of 0.013–0.018 *z*_*R*_). These were obtained in the previous studies of external SHG and coherent superposition of two LG modes works.

These results clearly indicate that the bottle beam generated by our proposed method provides a high potential barrier, which will be potentially used as a strong optical trap for light-absorptive particles^8^. Further investigation of optical forces on this bottle beam will be necessary to exploit numerous applications in the future^27,28^.

**Figure 3:**
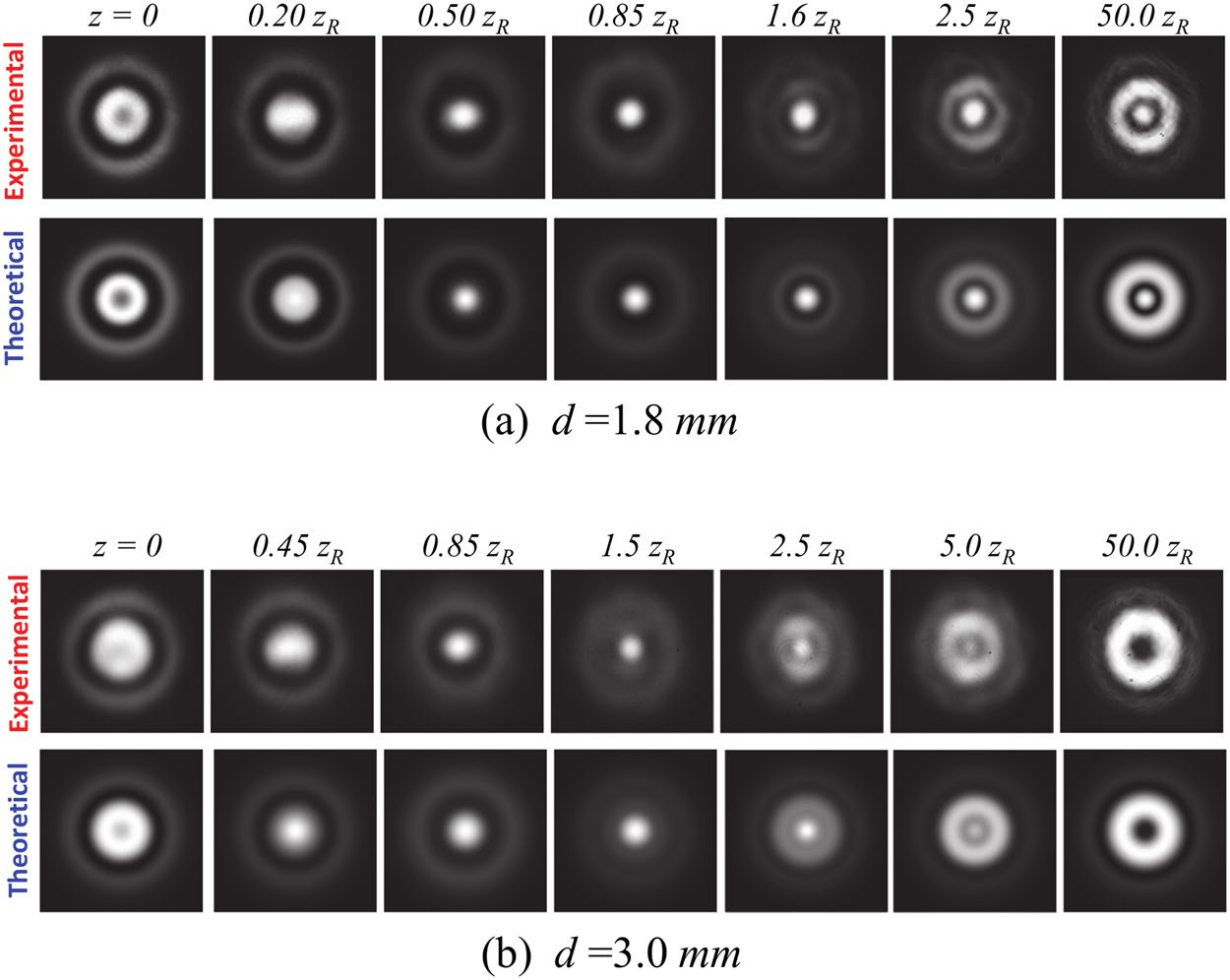
Experimental (upper row) and calculated (lower row) transverse spatial forms of the second-harmonic lasing mode at different longitudinal positions for (**a**) *d* = 1.8 mm and (**b**) *d* = 3.0 mm.

Figure 3(a,b) depict that the output generated from the system both experimentally and theoretically exhibits only a dark core with a relatively low intensity contrast in the near or far field owing to ~*π*/4 dephasing between Φ_0,*N*_ and Φ_1, *N*−1_, thereby losing the physical properties of a bottle beam. The value of *d* is then measured to be 1.8 mm or 3.0 mm, which indicates that the nonlinear crystal should be placed sufficiently close to the flat output coupler for bottle beam generation.

## Conclusions

We have successfully demonstrated the generation of a green bottle beam from a tightly pumped frequency-doubled Nd:YVO_4_ laser with a nearly hemispherical cavity. The bottle beam exhibited a well-isolated and narrower 3D dark core (high potential barrier) surrounded by a more symmetric and narrower potential wall, as compared with those described in previous publications^16,24^. Furthermore, the experimental beam propagation of the generated bottle beam was in good agreement with the results of a numerical analysis based on the superposition of radial LG modes with different radial indices.

## Methods

**Figure 4:**
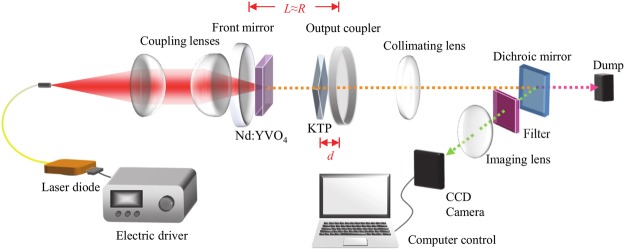
Experimental setup for a bottle beam laser resonator with a nearly hemispherical cavity configuration and intracavity SHG.

Figure 4 shows the experimental setup for our bottle beam laser resonator. The laser cavity consisted of a 30-mm radius-of-curvature concave input mirror with high reflectivity for 1064 nm (*R* > 99.8%) and high transmissivity (*T* > 95%) for 808 nm, and a flat output coupler with high reflectivity (*R* = 98%) for 1064 nm. The optical cavity length was fixed to be approximately 29.2 mm, so as to establish a nearly hemispherical resonator. An *a*-cut 2.0 at.% Nd:YVO_4_ crystal with a thickness of 2 mm and an aperture of 10 × 10 mm^2^ was used, and its surfaces were anti-reflection coated for 1064 nm (*R* < 0.2%). The crystal was wrapped with indium foil and mounted in a water-cooled copper holder to maintain the crystal temperature, and it was placed within a distance of ca. 1 mm from the concave input mirror. With this system, the fundamental cavity mode radius was estimated to be ca. 240 μm. A 3.0 W 808-nm fiber-coupled laser diode (core diameter: 100 μm, numerical aperture: 0.16) was used as a pump source, and its output was tightly focused to a spot size of radius 25 μm onto the crystal, so as to ensure a tightly-pumping condition, as reported in a previous publication^23^.

## Electronic supplementary material

Supplementary Information
