# Supplementary material for: Bottle beam generation from a frequency-doubled Nd:YVO4 laser
Source: Sci Rep. 2018 Nov 8;8:16576. doi: 10.1038/s41598-018-34783-z (PMC6224436; doi:10.1038/s41598-018-34783-z)
Supplement: Supplementary file 1 — Supplementary Information [file 41598_2018_34783_MOESM1_ESM.docx]

**Title**

Bottle beam generation from a frequency-doubled Nd:YVO_4_ laser

**Author names**

J. C. Tung,^1,2,+^ Y. Y. Ma,^1,+^ K. Miyamoto,^1,2^ Y. F. Chen,^3^ and T. OMATSU^1,2,*^

**Affiliations**

^1^Graduate School of Engineering, Chiba University, 1-33 Yayoi-cho, Inage-ku, Chiba 263-8522, Japan

^2^Molecular Chirality Research Center, Chiba University, 1-33, Yayoi-cho, Inage-ku, Chiba 263-8522, Japan

^3^Department of Electrophysics, National Chiao Tung University, 1001, Ta-Hsueh Rd., Hsinchu 30010, Taiwan

^+^ Equally contributed to this work

*Correspondence to omatsu@faculty.chiba-u.jp (Takashige Omatsu)

Phone number +81-43-290-3477, FAX +81-43-290-3490

jungchen_tung@chiba-u.jp (J. C. Tung)

axga0890@chiba-u.jp (Y. Y. Ma)

k-miyamoto@faculty.chiba-u.jp (K. Miyamoto)

yfchen@cc.nctu.edu.tw (Y. F. Chen)

**Supplementary Information**

The second-harmonic electric field with *p*=1 is given by

.

(1)

Here,

,

(2)

,

(3)

, and

(4)

.

(5)

The relationships

$L _{0}(x)^{ 2}=L _{0}(x)$,

(6)

$L _{0}(x)L _{1}(x)=L _{1}(x)$,

(7)

$L _{1}(x)^{ 2}=2L _{2}(x)-2L _{1}(x)+L _{0}(x)$,

(8)

and

$$L_{u}\left(x\right)=\frac{1}{2^{u}}\sum_{m=0}^{u} \frac{u!}{(u-m)!m!}L_{u-m}\left(2x\right)$$

(9)

are then used. The beam parameters (mode field radius and wavenumber) of the second harmonics are further given by $w'_{o}=w_{o}/\sqrt{2}$ and $k^{'}=2k$.

The second-harmonic lasing mode can, thus, be expressed by

,

(10)

where , $b_{1}=a_{0} a_{1}$, and $b_{2}=a_{1}^{2}/2$.
